# Bioanalysis of amphetamines in alternative matrices using a sensitive and validated liquid chromatography-tandem mass spectrometry method and its application to real samples

**DOI:** 10.1038/s41598-025-30861-1

**Published:** 2026-03-07

**Authors:** Humera Shafi Makhdoom, Ali Imran Abid, Nadeem Ul Hassan Khan, Zeerak Abbas

**Affiliations:** 1 Clinical and Forensic Toxicology Department, Chughtai Healthcare, Lahore, Pakistan; 2 Department of Pharmacy, University of Lahore, Lahore, Pakistan

**Keywords:** Amphetamines, UHPLC-ESI-MS/MS, Biological matrices, Toxicology, Validation, Biological techniques, Biomarkers, Chemistry, Environmental sciences, Medical research

## Abstract

Amphetamines abuse remains a major concern in both clinical and forensic toxicology. While conventional matrices like blood and urine are commonly used, their detection window is limited. This study aimed to validate a UHPLC-ESI-MS/MS method for amphetamines detection across both conventional and alternative biological specimens and apply it to real patient samples. A validated UHPLC-ESI-MS/MS method was used to quantify amphetamines in whole blood, urine, oral fluid, scalp hair, and fingernail specimens collected from fifty amphetamine abusers. Method validation followed international guidelines, assessing parameters including linearity, sensitivity, precision, and carry-over. Decontamination protocols and wash analyses were performed to exclude external contamination for keratinized matrices. The method demonstrated excellent linearity (r² = 0.995–0.998) and sensitivity (LOD = 2 ng/mL or ng/mg) across all matrices. Oral fluid showed strong correlation with recent drug use and was a reliable non-invasive alternative to blood and urine. Hair yielded the highest amphetamine concentrations due to melanin binding, whereas nail showed lower levels but provided a complementary window for retrospective exposure. Simultaneous quantification of amphetamines in five matrices provides a comprehensive assessment of both recent and chronic drug use. Hair remains the superior matrix for long-term detection, but nail offers valuable supplementary information. Oral fluid emerged as a practical and reliable tool for recent exposure monitoring. This is the first study to report amphetamine concentrations in all five matrices from the same individuals, offering critical insights for toxicological interpretations.

## Introduction

Amphetamines are a class of sympathomimetic phenethylamines characterized by potent central nervous system (CNS) stimulant effects^1^. While they are legitimately prescribed for conditions such as narcolepsy, attention-deficit/hyperactivity disorder (ADHD), and obesity^2^, their high potential for abuse has made them a persistent concern in both clinical and forensic toxicology. Common pharmaceutical forms include tablets, sustained-release capsules, and elixirs, yet amphetamine (AMP), methamphetamine (METH), and 3,4-methylenedioxymethamphetamine (MDMA) are also widely misused for recreational and performance-enhancing purposes^3^. Methylenedioxyamphetamine (MDA), a metabolite of MDMA, further expands this category of synthetic amphetamines.

Due to their strong psychoactive properties, these compounds are frequently abused by individuals seeking increased alertness, endurance, or euphoria, making them particularly attractive in competitive environments. Consequently, amphetamines are listed among prohibited substances by organizations such as the International Olympic Committee Medical Commission. Chronic abuse is associated with significant neurotoxicity, especially to dopamine and serotonin systems^4^, underscoring the need for reliable detection methods in biological specimens for both therapeutic monitoring and forensic investigation.

Despite the recent surge in new psychoactive substances (NPS), amphetamine-type stimulants (ATS) remain a dominant concern globally. According to the 2022 World Drug Report by the United Nations Office on Drugs and Crime (UNODC), amphetamines continue to show an alarming rise in global use over the past decade^5^. Methamphetamine is notable for its high oral bioavailability (~ 67%), extensive tissue distribution (3–7 L/kg), and long elimination half-life (~ 10–12 h depending on route of administration). Its substantial unmetabolized excretion (approximately 45% in urine within 24 h) and partial conversion to amphetamine highlight the importance of urine analysis in monitoring use^6,7^. Toxic and fatal concentration ranges for methamphetamine in various tissues have been reported, reinforcing the urgency for accurate and sensitive detection across matrices.

Traditionally, amphetamines have been detected using immunoassay screening methods followed by confirmatory techniques such as gas chromatography-mass spectrometry (GC-MS)^8^. While GC-MS remains a gold standard due to its high selectivity, its reliance on derivatization increases both cost and turnaround time. More recently, liquid chromatography–tandem mass spectrometry (LC-MS/MS) has gained traction due to its superior sensitivity, faster analysis, and minimal sample preparation^9^. However, LC-MS/MS methods are highly susceptible to matrix effects, such as ion suppression or enhancement^10^, necessitating thorough validation procedures to ensure analytical robustness and reproducibility.

Conventional biological matrices such as blood and urine are invaluable for assessing recent drug exposure^11^. Yet, alternative matrices like oral fluid, hair, and nails, owing to their ease of collection, non-invasiveness, and longer detection windows, are increasingly recognized for their utility in drug testing^12^. Oral fluid is particularly reflective of free drug levels in plasma, making it suitable for evaluating recent use^13^. Meanwhile, keratinized matrices such as hair and nails offer extended detection windows, preserving a record of drug intake over weeks to months^14,15^.

While numerous studies have investigated amphetamines in individual matrices^16–18^, there remains a significant gap in comprehensive analyses comparing both conventional and alternative biological specimens collected simultaneously from the same subjects. Addressing this gap, the present study aims to develop and validate a rapid and sensitive ultra-high-performance liquid chromatography–electrospray ionization tandem mass spectrometry (UHPLC-ESI-MS/MS) method for the simultaneous quantification of amphetamines in blood, urine, oral fluid, scalp hair, and fingernails. By examining these diverse matrices in parallel from the same individuals with known histories of amphetamine abuse, this research provides critical insight into detection windows, drug incorporation mechanisms, and the comparative value of each matrix. Such comprehensive data are especially relevant in forensic contexts, including post-mortem investigations or cases with limited sample availability, such as exhumations.

## Experimental work

### Materials and methods

A certified reference standard mixture (Mixture-6) containing Amphetamine (AMP), Methamphetamine (METH), 3,4-Methylenedioxymethamphetamine (MDMA), and 3,4-Methylenedioxyamphetamine (MDA), each at a concentration of 250 µg/mL, was obtained from Cerilliant (Round Rock, TX, USA), along with a deuterated internal standard, Amphetamine-d6 (1 mg/mL). Agilent Mega Bond Elute 6 mL solid-phase extraction (SPE) cartridges were sourced from HA Shah and Sons, Pakistan. Reagents including monobasic sodium phosphate, ammonium hydroxide, glacial acetic acid, hexane, dichloromethane, isopropanol, methanol, and hydrochloric acid (all ACS grade) were procured from Merck (Germany). LC-MS grade solvents, methanol, acetonitrile, water, and formic acid were also supplied by Merck. Plasticware and other consumables were purchased from Fisher Scientific (Pittsburgh, PA, USA).

Working standard solutions were prepared by diluting the amphetamines mixture and Amphetamine-d6 separately in methanol to achieve final concentrations of 2500 ng/mL and 100 ng/mL, respectively. These solutions were used to prepare calibration standards at concentrations of 2, 5, 10, 25, 50, 100, 250, 500, 750, 1000, 1500, and 2000 ng/mL (or ng/mg for hair and nail samples), for method validation purposes.

Chromatographic separation was carried out on an Agilent Poroshell 120 EC-C18 analytical column (2.1 mm × 50 mm, 2.7 μm particle size) maintained at 40 °C. Quantitative analysis was performed using an Agilent 1260 Infinity UHPLC system coupled to an Agilent 6470 triple quadrupole mass spectrometer. The mobile phases used for gradient elution were LC-MS grade water with 0.1% formic acid (mobile phase A) and LC-MS grade methanol with 0.1% formic acid (mobile phase B). The flow rate was set at 0.5 mL/min, with the gradient program as follows: 0.0 min – 15% B; 0.2 min – 15% B; 3.0 min – 90% B; 4.0 min – 15% B. The injection volume was 2 µL, with a total run time of 4.0 min and a post-run time of 2.0 min.

Electrospray ionization (ESI) in positive polarity mode, enhanced by Agilent Jet Stream (AJS) technology, was employed with the following parameters: delta EMV + 200, sheath gas temperature 350 °C, gas flow 12 L/min, nebulizer pressure 50 psi, and capillary voltage 2500 V. Compound-specific ionization and fragmentation parameters are detailed in Table 1, including selected precursor and product ions.

Data acquisition, instrument control, and processing were performed using MassHunter version 1.6.3 software. Detection and quantification were conducted in multiple reaction monitoring (MRM) mode, with two MRM transitions selected per analyte and one for the internal standard. Analyte quantification was based on the peak area ratio relative to the internal standard. Each compound underwent individual parameter optimization using the Agilent Optimizer tool, confirming that positive ionization mode yielded superior sensitivity for the target analytes.

**Table 1:** Tandem mass spectrometer settings and retention times for amphetamines and internal standard.

Compound	Precursor ion	Product ion	Fragmentor voltage (V)	Collision energy (V)	Cell acceleration voltage (V)	Dwell time (ms)
Amphetamine	136.1	119.1	66	5	7	100
		91		17		
Methamphetamine	150.1	65.1	92	45		
		91		17		
MDMA	194.1	163	97	9		
		105		25		
MDA	180.1	163	61	5		
		105		21		
Amphetamine-d6 (IS)	142.1	93.1	66	13		

### Real samples collection, preservation and storage

Sample collection, preservation, and storage followed established recommendations consistent with EWDTS and SAMHSA guidelines. Blood was obtained via standardized venipuncture into gray-top tubes containing sodium fluoride, with immediate stabilization, labelling, refrigeration, and full chain-of-custody documentation. Urine specimens were collected in sterile, tamper-evident 50 mL containers, with temperature verification, adulteration checks, and prompt refrigeration to prevent degradation or substitution. Oral fluid was collected using validated devices under controlled conditions; participants abstained from eating, drinking, smoking, or placing objects in the mouth for at least 30 min prior to collection, after which they expectorated into a 50 mL cup pre-filled with sodium fluoride^19^. Scalp hair was sampled from the posterior vertex region by cutting a pencil-thick tuft close to the scalp, with root-end alignment, documentation of length and orientation, and storage in paper envelopes; the proximal 3–5 mm segment was used for analysis following standard decontamination procedures^20^. Fingernail samples were obtained by clipping 2–3 mm from the distal edges of all ten fingers, cleaned to remove surface debris, labelled by digit, and stored in paper envelopes at room temperature until analysis^21^.

Drug-free biological matrices, including blood, urine, oral fluid, scalp hair, and fingernails were voluntarily donated by laboratory personnel following informed consent. All matrices were confirmed negative for drugs and subsequently used as negative control materials.

The study cohort included 50 individuals with a documented history of amphetamine use. Participants with poly-drug abuse or recent cosmetic treatment of hair or nails were excluded to avoid confounding factors. Eligible participants were between 13 and 65 years old, with a history of amphetamine use for at least the preceding three months and confirmed recent exposure within 36 h. Informed consent was obtained from all participants before sample collection, which occurred prior to the initiation of treatment for substance-use disorder. Demographic information and detailed amphetamine-use histories were recorded for each participant.

### Sample pre-treatment

Blood, urine, and oral fluid samples were analyzed directly without any pre-treatment or dilution. Scalp hair and fingernail specimens underwent a decontamination procedure to remove potential external contaminants. Samples were first washed with deionized water, followed by two sequential washes with 5 mL of dichloromethane at room temperature, each lasting 5 min. Between washes, samples were gently dried using absorbent paper to prevent cross-contamination and ensure thorough cleaning.

The dichloromethane extracts were collected, transferred to clean tubes, evaporated to dryness using a nitrogen evaporator at 40 °C, and reconstituted in 500 µL of acetonitrile. These reconstituted extracts were stored at 4 °C and later analyzed to assess the presence of external contamination.

For extraction, 25 mg of the cleaned and dried hair or nail samples were finely chopped and incubated overnight in 1 mL of 1 N sodium hydroxide at room temperature to achieve complete digestion. Negative control specimens (drug-free hair and nails) were subjected to the same digestion protocol for validation purposes.

### Toxicological analysis

Aliquots of 1 mL from each calibrator, control, and real sample were transferred into labeled 15 mL polypropylene tubes. Subsequently, 25 µL of the working internal standard solution and 3 mL of 0.1 M phosphate buffer (pH 6.0) were added to each tube. The mixtures were centrifuged at 4000 rpm for 15 min to separate any particulates. The supernatants were then carefully transferred onto solid-phase extraction (SPE) columns.

Prior to sample loading, SPE columns were conditioned sequentially with 3 mL of methanol, followed by 3 mL of deionized water, and 2 mL of 0.1 M phosphate buffer (pH 6.0). After sample application, the columns were washed successively with 3 mL each of deionized water, 0.5 M acetic acid, and methanol to remove residual impurities.

Analytes were eluted using 3 mL of a freshly prepared mixture of methylene chloride, isopropanol, and ammonium hydroxide in a ratio of 78:20:2 (v/v). To stabilize the analytes, 100 µL of 0.1 N hydrochloric acid was added to the organic extracts. The eluates were then evaporated to dryness under a stream of nitrogen at 40 °C.

The dried residues were reconstituted in 150 µL of acetonitrile containing 0.1% formic acid, vortex-mixed, and transferred into autosampler vials equipped with 250 µL glass inserts. Vials were capped and stored appropriately until analysis by LC-MS/MS.

### Method validation

The analytical method was validated in accordance with international guidelines, including those provided by scientific working group for forensic toxicology (SWGTOX) (SWGTOX, 2013)^22^ and other relevant regulatory standards. A seven-point calibration curve was constructed using linear regression analysis of the analyte-to-internal standard peak area ratios. Calibration standards at concentrations of 5, 10, 50, 100, 500, 1000, and 2000 ng/mL (or ng/mg for hair and nail) were prepared in triplicate and analyzed over five consecutive days in each biological matrix (blood, urine, oral fluid, hair, and nails) to assess linearity and matrix effects.

Matrix-matched calibration curves were generated for each matrix to evaluate potential matrix-dependent variability. For each calibration batch, a blank matrix and a blank matrix containing only the internal standard were included as quality controls but were excluded from the calibration curve construction.

Accuracy (expressed as recovery) and precision (intra- and inter-assay variability) were evaluated at three concentration levels: 25, 250, and 1500 ng/mL. Each level was analyzed in triplicate over five consecutive days. Relative standard deviation (RSD) and bias were calculated to assess method performance.

The limit of detection (LOD) was defined as the lowest concentration with a signal-to-noise ratio (S/N) greater than 3, and the lower limit of quantification (LLOQ) was defined as the lowest concentration with S/*N* ≥ 10, along with acceptable precision and accuracy.

Ion suppression and enhancement were assessed according to SWGTOX guidelines (SWGTOX, 2013), using post-extraction addition methods to evaluate potential matrix effects on electrospray ionization efficiency.

Dilution integrity was tested using spiked samples at concentrations 10-fold and 50-fold higher than the upper limit of quantification. These samples were diluted in mobile phase prior to sample preparation, and results were evaluated for accuracy and precision.

Carry-over was assessed by injecting blank matrix samples immediately after the highest calibration standard (*n* = 5). No significant carry-over was observed if the analyte response in the blank was less than 20% of the LLOQ.

Stability testing was conducted by reanalysing stored (archival) samples to ensure consistency of results over time under defined storage conditions.

**Fig. 1:**
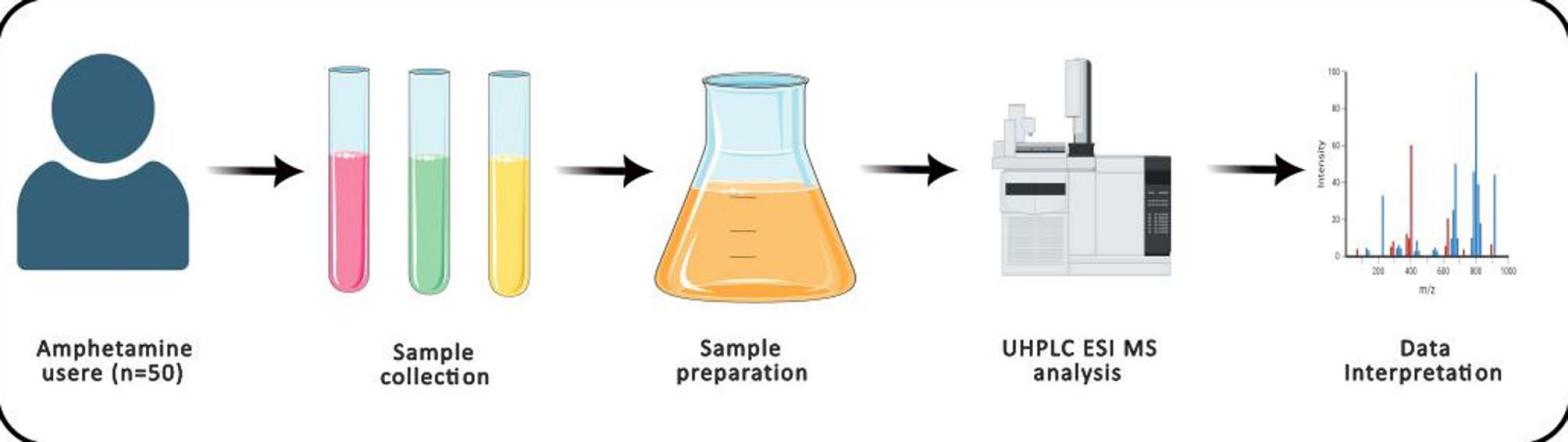
Schematic illustrating the experimental workflow, from sample collection to data interpretation.

A summary of the main steps involved in the experimental work is illustrated in Fig. 1.

## Results

### Method validation results

Linearity was assessed by plotting the average relative response (analyte/internal standard peak area ratio) of each calibrator against its corresponding concentration. Linear regression analysis was applied, and the correlation coefficient (r²) was used to evaluate the linearity of each calibration curve. In accordance with toxicology quality assurance standards, a minimum correlation coefficient of 0.990 was deemed acceptable. The developed method demonstrated excellent linearity across all tested matrices—blood, urine, oral fluid, hair, and nail, up to 2000 ng/mL (or ng/mg for hair and nail), with correlation coefficients ranging from 0.995 to 0.998.

The limits of detection (LOD) and quantification (LLOQ) for amphetamines were determined to be 2 ng/mL and 5 ng/mL (or ng/mg), respectively, for all biological matrices. The dilution integrity study confirmed that precision and accuracy remained within ± 15% of nominal values for samples diluted up to 50-fold above the upper calibration range.

**Fig. 2:**
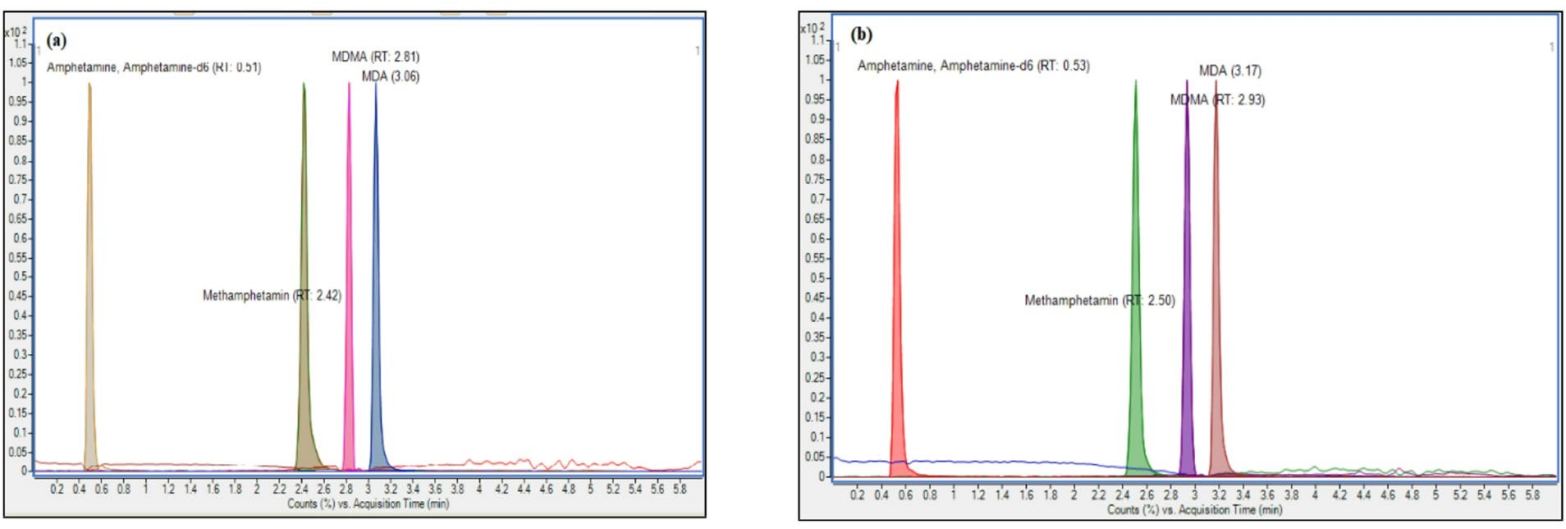
Representative overlaid extracted ion chromatograms of amphetamines MRMs in (**a**) scalp hair and (**b**) finger nail matrices (25ng/mg).

No interfering peaks from endogenous substances or carry-over effects were observed in any of the matrices, confirming the method’s specificity. Representative extracted ion chromatograms of amphetamines in hair and nail matrices at 25 ng/mg are presented in Fig. 2.

The validation results are summarized in Table 2.

**Table 2:** LC-MS/MS method validation results for amphetamines in biological matrices.

Parameter			Blood		Urine		Oral fluid		Scalp hair		Finger nail
Amphetamine											
Mean intra-assay precision (*n* = 45, RSD %)			2.5		4.0		3.4		2.4		4.3
Mean inter-assay precision (*n* = 45, RSD %)			4.2		3.3		4.3		4.9		5.0
Accuracy (*n* = 45, ± %)			−1.1		−0.3		−2.0		−8.5		−6.7
Mean extraction recovery (*n* = 45, %)			98.9		99.7		98.0		91.5		93.3
Coefficient of linearity (r^2^)			0.997		0.998		0.997		0.996		0.995
Ion suppression/enhancement (± %)			−6.2		−4.7		−6.9		−11.8		−14.4
Methamphetamine											
Mean intra-assay precision (*n* = 45, RSD %)			2.1		2.4		2.9		4.8		5.9
Mean inter-assay precision (*n* = 45, RSD %)			4.8		3.9		3.8		4.0		7.1
Accuracy (*n* = 45, ± %)			−0.8		−0.2		−1.4		−7.7		−8.1
Mean extraction recovery (*n* = 45, %)			99.2		99.8		98.6		92.3		91.9
Coefficient of linearity (r^2^)			0.996		0.998		0.996		0.995		0.996
Ion suppression/enhancement (± %)			−5.7		−1.9		−4.0		−10.2		−11.2
MDMA											
Mean intra-assay precision (*n* = 45, RSD %)			2.2		2.1		3.2		3.6		5.3
Mean inter-assay precision (*n* = 45, RSD %)			4.5		4.9		3.7		2.9		7.6
Accuracy (*n* = 45, ± %)			−2.5		−0.9		−5.6		−8.2		−8.4
Mean extraction recovery (*n* = 45, %)			97.5		99.1		94.4		91.8		91.6
Coefficient of linearity (r^2^)			0.997		0.998		0.996		0.997		0.995
Ion suppression/enhancement (± %)			−6.4		−5.0		−6.1		−7.3		−8.2
MDA											
Mean intra-assay precision (*n* = 45, RSD %)			3.3		3.8		4.2		5.2		5.7
Mean inter-assay precision (*n* = 45, RSD %)			6.1		5.6		4.9		5.8		4.9
Accuracy (*n* = 45, ± %)			−0.7		−0.4		−3.2		−6.8		−8.9
Mean extraction recovery (*n* = 45, %)			99.3		99.6		96.8		93.2		91.1
Coefficient of linearity (r^2^)			0.997		0.998		0.996		0.997		0.995
Ion suppression/enhancement (± %)			−7.3		−2.7		−10.4		−5.9		−9.1

### Real samples analysis results

Amphetamines were quantitatively analyzed in both conventional (blood, urine, oral fluid) and alternative (scalp hair, fingernails) biological specimens collected from fifty patients. The mean concentrations of amphetamines detected in each matrix are summarized in Table 3.

**Table 3:** Concentration of amphetamines in conventional and alternative patient specimens.

History (Chronic/ acute)	No. of patients	Drug/metabolite	Blood (ng/mL)	Urine (ng/mL)	Oral fluid (ng/mL)	Scalp hair (ng/mg)	Finger nail (ng/mg)
≥ 1 to ˂3 mo/36 h	6	Amphetamine	35–98	57–230	19–39	7–18	5–11
	6	Methamphetamine	193–1580	121–560	26–121	9–21	7–19
	4	MDMA	249–1674	153–446	78–313	6–15	10–11
	4	MDA	65–147	68–336	35–78	Not detected	Not detected
≥ 3 to ˂6 mo/36 h	13	Amphetamine	66–87	76–107	35–58	13–29	12–33
	13	Methamphetamine	89–1351	97–1106	46–310	11–19	9–16
	7	MDMA	350–1168	165–1737	41–98	8–33	10–25
	7	MDA	45–99	73–150	15–69	5–6	Not detected
≥ 6 to ˂9 mo/36 h	9	Amphetamine	87–149	59–348	49–161	25–62	33–45
	9	Methamphetamine	231–992	93–1279	105–219	17–38	13–28
	11	MDMA	171–1378	54–1063	90–365	41–69	27–55
	11	MDA	41–53	57–211	38–91	7–11	5–6

*mo* Months, *h* Hours.

## Discussion

This study presents a robust, sensitive, and rapid UHPLC-ESI-MS/MS method for the quantification of amphetamines in both conventional (blood, urine, oral fluid) and alternative (scalp hair, fingernail) biological matrices. Following rigorous validation in line with international guidelines, the method was successfully applied to specimens collected from fifty individuals with a history of amphetamine abuse.

While blood and urine are the most commonly used matrices in clinical and forensic toxicology, their utility is limited to detecting recent drug intake^23,24^. Amphetamines are rapidly metabolized and excreted^25^, making these matrices effective only within a short post-consumption window. Oral fluid has gained attention as an alternative matrix due to its non-invasive collection and its capacity to reflect the free (pharmacologically active) fraction of drugs^26^, closely correlating with blood concentrations. In this study, oral fluid was collected via expectoration—a method that, while potentially uncomfortable, yielded representative concentrations of amphetamines. Based on the findings, oral fluid proved to be a promising matrix for assessing recent amphetamine intake, offering advantages over blood and urine in terms of ease of collection and reduced risk of adulteration. Unlike blood collection, which requires trained personnel and carries risks of infection or sample contamination^27,28^, oral fluid collection can be performed easily and safely without specialized equipment. Furthermore, oral fluid sampling minimizes privacy concerns and is less susceptible to adulteration or substitution, common issues associated with urine testing.

The results of this study support the use of oral fluid as a reliable and convenient biological matrix for the detection of recent amphetamine use. Its strong correlation with plasma concentrations, coupled with its ease of collection and lower risk of tampering, makes it particularly valuable for applications in roadside drug testing, workplace monitoring, and forensic toxicology settings.

The use of keratinized matrices, particularly hair and nails, for retrospective drug monitoring has expanded significantly in recent years^14^. Hair analysis is well-established and offers unique advantages, such as non-invasive collection, high stability, and the capacity to provide a long detection window^29^. Drugs can persist in hair for extended periods, and when segmented, hair analysis can reveal a historical pattern of drug use^30^. Drug incorporation into hair occurs via multiple routes, including systemic circulation, sweat, and sebum^31,32^. However, one of the major analytical challenges is external contamination. To address this, the current study implemented a rigorous decontamination protocol using dichloromethane, and the wash solutions were analyzed to assess potential environmental exposure. The absence of amphetamines in all hair washes indicated that the detected drug levels were due to actual ingestion, not external contamination. This approach is aligned with recommendations from the Society of Hair Testing (SOHT), reinforcing the reliability of the hair results.

Similarly, fingernails have emerged as a viable matrix for evaluating long-term drug exposure^33^. Drug incorporation into nails occurs both from systemic blood flow at the nail matrix and from the nail bed during plate growth^34^. Although nail analysis is less explored compared to hair^35^, it offers comparable advantages, non-invasive collection, resistance to adulteration, and an extended detection window^36^. However, unlike hair, nails lack melanin, which has been shown to bind basic drugs like amphetamines^15^. This difference likely contributes to the generally lower concentrations observed in nail compared to hair. Our findings support this, with scalp hair consistently exhibiting higher amphetamine concentrations than fingernails across the study cohort.

Growth rate and sampling site further influence drug concentrations in these matrices. Scalp hair grows at approximately 1 cm/month^37^, and the 3 cm proximal segment analyzed in this study reflects roughly three months of drug exposure. In contrast, fingernails grow at about 3 mm/month^38^. The 1–2 mm clippings taken from the distal edge of the nail represent exposure from a period approximately 3–5 months prior, depending on individual nail growth rates. This temporal mismatch should be carefully considered when interpreting comparative results between these two matrices.

Importantly, our data showed a strong concordance between reported amphetamine use history and measured drug concentrations in both hair and nail. Moreover, a clear trend of increasing amphetamine levels with longer reported durations of use was observed. These findings confirm the potential of both matrices for monitoring chronic amphetamine exposure and suggest that nails may serve as a complementary matrix to hair. Nonetheless, the establishment of matrix-specific limits of detection (LOD) and quantification (LOQ) is essential for accurate interpretation in nail testing.

It should be noted that, as per the EWDTS guidelines, the cutoff for amphetamines and their derivatives in hair is set at 0.2 ng/mg. The analytical method employed in this study approaches, but does not fully reach, this cutoff. Consequently, while the method is reliable for detecting moderate to heavy amphetamine use, there remains a possibility of false negatives in cases of low or occasional exposure. This limitation has been acknowledged to provide context for interpreting hair analysis results and should be taken into consideration when evaluating the findings. Future work may focus on improving method sensitivity to reliably detect lower-level exposure, if feasible.

To our knowledge, this is the first study to simultaneously quantify amphetamines in blood, urine, oral fluid, scalp hair, and fingernails from the same individuals at a single time point. By providing a comprehensive, multi-matrix comparison, our findings offer valuable insights into the dynamics of drug distribution and accumulation across different biological specimens. This approach not only captures recent use through fluid matrices such as blood, urine, and oral fluid but also reflects long-term exposure via hair and nails, offering a holistic view of consumption patterns. Such an integrated perspective enhances the applicability of these results in both clinical and forensic toxicology, informing more accurate interpretation of drug intake. Furthermore, this multi-matrix methodology holds considerable potential for advancing monitoring programs, early intervention strategies, and evidence-based decision-making in substance use management.our knowledge, this is the first study to simultaneously quantify amphetamines in blood, urine, oral fluid, scalp hair, and fingernails from the same individuals at a single time point. The comprehensive comparison across matrices enhances the utility of these findings in both clinical and forensic toxicology contexts. This multi-matrix approach enables a more complete understanding of drug exposure patterns, capturing both recent use and long-term consumption and holds significant promise for advancing substance use monitoring and intervention strategies.

## Conclusion

This study successfully developed and validated a sensitive, rapid, and robust UHPLC-ESI-MS/MS method for the simultaneous quantification of amphetamines in both conventional (blood, urine) and alternative (oral fluid, scalp hair, fingernail) biological specimens. The method demonstrated excellent linearity, precision, accuracy, and minimal matrix interference across all tested matrices, complying with international validation guidelines. Application of the method to real samples from amphetamine abusers revealed important insights into the distribution of amphetamines across different biological compartments.

Among the tested matrices, oral fluid emerged as a promising alternative to blood and urine for detecting recent amphetamine exposure due to its ease of collection and strong correlation with active drug levels. Keratinized matrices, particularly hair and fingernail, proved valuable for retrospective monitoring of chronic drug use, with hair generally showing higher amphetamine concentrations, likely due to melanin binding and faster drug incorporation. Nonetheless, fingernails also reflected prolonged exposure, offering an effective alternative when hair is unavailable or compromised.

To our knowledge, this is the first study to simultaneously evaluate amphetamines in five distinct matrices from the same cohort of individuals. The findings provide a multidimensional understanding of drug detection windows, drug incorporation mechanisms, and matrix-specific characteristics, offering a comprehensive framework for both clinical and forensic toxicological applications. This method and its insights can significantly aid in interpreting drug use patterns, particularly in challenging scenarios such as legal investigations, post-mortem toxicology, and cases involving limited sample availability.

## Data Availability

All data generated or analyzed during this study are included in this published article.

## Abbreviations

SPE: Solid-phase extraction
LOD: Limits of detection
LOQ: Limits of quantification
SOHT: Society of hair testing
ESI: Electrospray ionization
EWDTS: European workplace drug testing society
AMP: Amphetamine
METH: Methamphetamine
MDMA: 3,4-methylenedioxymethamphetamine
MDA: 3,4-methylenedioxyamphetamine
ADHD: Attention-deficit/hyperactivity disorder
CNS: Central nervous system
MRM: Multiple reaction monitoring
UHPLC-ESI-MS/MS: Ultra-high-performance liquid chromatography–electrospray ionization tandem mass spectrometry
LLOQ: Lower limit of quantification
RSD: Relative standard deviation
UNODC: United Nations Office on Drugs and Crime
